# Stimulated thyroglobulin and pre-ablation antithyroglobulin antibody products can predict the response to radioiodine therapy of TgAb-positive differentiated thyroid cancer patients: a retrospective study

**DOI:** 10.3389/fendo.2023.1222470

**Published:** 2023-09-22

**Authors:** Na Han, Chenghui Lu, Jiao Li, Congcong Wang, Zilong Zhao, Yingying Zhang, Xinfeng Liu, Zengmei Si, Guoqiang Wang, Zenghua Wang, Fengqi Li, Xufu Wang

**Affiliations:** ^1^ Department of Nuclear Medicine, The Affiliated Hospital of Qingdao University, Qingdao, Shandong, China; ^2^ Department of Nuclear Medicine, Ordos Central Hospital, Ordos, Inner Mongolia, China

**Keywords:** differentiated thyroid cancer, serum antithyroglobulin antibody, radioiodine therapy, response to therapy, prognosis

## Abstract

**Objective:**

We aimed to explore the predictive value of stimulated thyroglobulin (sTg) and pre-ablation antithyroglobulin (pa-TgAb) products for the effect of radioiodine therapy (RAIT) on TgAb-positive differentiated thyroid cancer (DTC) patients.

**Methods:**

In this study, we enrolled 265 patients with TgAb-positive DTC who underwent RAIT after total thyroidectomy (TT). Based on the last follow-up result, the patients were divided into two groups: the excellent response (ER) group and the non-excellent response (NER) group. We analyzed the factors related to the effect of RAIT.

**Results:**

The ER group consisted of 197 patients. The NER group consisted of 68 patients. For the univariate analysis, we found that the maximal tumor diameter, whether with extrathyroidal extension (ETE), bilateral or unilateral primary lesion, multifocality, preoperative TgAb (preop-TgAb), pa-TgAb, sTg × pa-TgAb, initial RAIT dose, N stage, and surgical extent (modified radical neck dissection or not), showed significant differences between the ER group and NER group (all *p*-values <0.05). The receiver operating characteristic (ROC) curves showed that the cutoff value was 724.25 IU/ml, 424.00 IU/ml, and 59.73 for preop-TgAb, pa-TgAb, and sTg × pa-TgAb, respectively. The multivariate logistic regression analysis results indicated that pa-TgAb, sTg × pa-TgAb, initial RAIT dose, and N stage were independent risk factors for NER (all *p*-values <0.05). For the Kaplan–Meier analysis of disease-free survival (DFS), the median DFS of the patients with sTg × pa-TgAb < 59.73 and initial RAIT dose ≤ 100 mCi was significantly longer than that of the patients with sTg × pa-TgAb ≥ 59.73 (50.27 months vs. 48.59 months, *p* = 0.041) and initial RAIT dose >100 mCi (50.50 months vs. 38.00 months, *p* = 0.030).

**Conclusion:**

We found the sTg and pa-TgAb conducts is a good predictor of the efficacy of RAIT in TgAb-positive DTC patients. It can play a very positive and important role in optimizing treatment, improving prognosis, and reducing the burden of patients.

## Introduction

Differentiated thyroid cancer (DTC) accounts for more than 95% of thyroid cancers and is the most common endocrine tumor. Surgery, selective radioiodine therapy (RAIT) after total thyroidectomy (TT), and thyroid-stimulating hormone (TSH) suppressive therapy are the standard treatments for DTC (1). As a specific tumor marker for long-term follow-up after RAIT in DTC, serum thyroglobulin (Tg) can predict DTC persistence, distant metastasis, or recurrence (2–4). However, the Tg level can be affected by the antithyroglobulin antibody (TgAb), which makes Tg measurement unreliable.

TgAb is mainly produced by lymphocytes, which react against thyroid self-antigens (5). Almost 25% of DTC patients are positive for circulating TgAb (6–8). In such patients, the value as a tumor marker of serum Tg will be substantially weakened since positive TgAb may result in false-negative results for DTC patients when it is measured with radioimmunoassay (9, 10). Therefore, as a considerable surrogate tumor marker for evaluating dynamic risk stratification, TgAb is adopted in the American Thyroid Association (ATA) guidelines (11).

In most previous studies, researchers mainly focused on the relationship between the time trend of TgAb after RAIT and the clinical outcome of DTC and revealed that the trend of TgAb was more important for prognosis in TgAb-positive DTC patients (12–16). However, it takes time to observe the time trend of TgAb. Therefore, identifying patients with a poor prognosis early is the focus in clinical practice. Pan et al. (17) reported a negative correlation between stimulated thyroglobulin (sTg) and TgAb, and their product could be used to predict the efficacy of RAIT and prognosis of DTC patients treated with RAIT. However, the research was mainly focused on TgAb-negative patients, and the sample size of TgAb-positive patients in their study was relatively small (88 patients). There is little other literature on the combination of sTg with pre-ablation TgAb (pa-TgAb) for predicting the effect of RAIT in TgAb-positive DTC patients. Thus, the purpose of our study was to explore the value of the product of sTg and pa-TgAb for predicting the curative effect of RAIT in TgAb-positive DTC patients.

## Materials and methods

### Study population

In this single-center retrospective study, we enrolled DTC patients with positive serum TgAb titers before the initial thyroidectomy and initial RAIT at the Department of Nuclear Medicine in the Affiliated Hospital of Qingdao University from January 2014 to September 2020.

The inclusion criteria were as follows (1) patients who received TT with neck dissection and were diagnosed with DTC by pathology; (2) patients with available data on sTg, preoperative TgAb (preop-TgAb), and pa-TgAb concentrations; (3) positive TgAb status with preop-TgAb ≥ 115 IU/ml and pa-TgAb ≥ 40 IU/ml (18); (4) sTg and pa-TgAb with substantial values; and (5) at least 24 months of follow-up after the initial RAIT.

The exclusion criteria included the following: (1) patients with partial thyroidectomy, (2) patients with undetectable sTg or pa-TgAb (below or above the detection limit), (3) patients with other malignant tumors, and (4) patients lost to follow-up.

### Clinicopathological variables and definitions

The following patients’ characteristics were analyzed: sex; age at diagnosis; maximal tumor diameter; multifocality; presence or absence of Hashimoto’s thyroiditis (HT); tumor with extrathyroidal extension (ETE); surgical extent (modified radical neck dissection or not) bilateral or unilateral primary lesion; tumor stage; lymph node metastasis (LNM) stage; risk stratification of recurrence; and preop-TgAb, sTg, and pa-TgAb measurements before the initial RAIT. TgAb and Tg were measured by using an electrochemiluminescence immunoassay (Roche, Switzerland).

Definitions: (1) Preop-TgAb was checked within 1 week prior to surgery. (2) sTg and pa-TgAb titers were measured within 2 days prior to the initial RAIT. (3) HT was confirmed by histopathological examination after surgery. (4) Bilaterality of the tumor was defined as a tumor that was located in both thyroid lobes. (5) The TNM stage and risk stratification of recurrence followed the American Joint Committee on Cancer (AJCC) TNM staging eighth edition (19) (**Table 1**) and 2015 ATA guidelines (11). (6) Disease-free survival (DFS) time was defined as there being no evidence of persistent disease since the initial RAIT. The end event was DTC recurrence or metastasis (18, 20, 21). (7) For each patient, the follow-up period was defined as the length of time from the initial RAIT and the last known patient contact, which was recorded by viewing the medical history or telephoning the patient. (8) Time trends in TgAb levels were calculated based on the following definitions (22): increase: elevation of TgAb levels greater than 50%; stability: TgAb levels fluctuate, rise, or decrease by less than 50%; and decrease: TgAb levels decrease by more than 50%.

**Table 1:** Staging guide for differentiated thyroid cancer (AJCC eighth edition) (19).

Age at diagnosis	T category	N category	M category	Stage
<55 years	Any T	Any N	M0	I
	Any T	Any N	M1	II
≥55 years	T1	N0/NX	M0	I
	T1	N1	M0	II
	T2	N0/NX	M0	I
	T2	N1	M0	II
	T3a/T3b	Any N	M0	II
	T4a	Any N	M0	III
	T4b	Any N	M0	IVA
	Any T	Any N	M1	IVB

T, tumor; N, node; M, metastasis.

### RAIT procedures and follow-up

The patients achieved the goal of TSH > 30 mU/L after thyroid hormone withdrawal and followed a low-iodine diet for 3–4 weeks. The RAIT dose was referred to as each patient’s specific extent of disease based on the 2015 ATA guidelines’ recommendations (11). Posttherapeutic whole-body scanning (Rx-WBS) and single-photon emission computed tomography/computed tomography (SPECT/CT) were performed 3–5 days after RAIT. After RAIT, levothyroxine treatment was maintained. The following follow-up data were recorded every 6–12 months: serum Tg, TgAb, diagnostic WBS (Dx-WBS), and neck ultrasound. In addition, the results of chest CT, ^18^F-FDG positron emission tomography/computed tomography (PET/CT), and fine needle aspiration (FNA) biopsy were also recorded if necessary.

### Responses to therapy assessments

The response to RAIT was divided into the following four categories according to serological measurements and imaging examinations (11, 22):

(1) Excellent response (ER): suppressed Tg < 0.2 ng/ml or sTg < 1 ng/ml with negative TgAb and the imaging results were negative
(2) Indeterminate response (IDR): suppressed Tg between 0.2 ng/ml and 1 ng/ml or sTg between 1 ng/ml and 10 ng/ml with negative TgAb or decreased or stable TgAb levels and the imaging results were negative
(3) Biochemical incomplete response (BIR): suppressed Tg > 1 ng/ml or sTg > 10 ng/ml with negative TgAb or increase in TgAb levels and the imaging results were negative
(4) Structural incomplete response (SIR): structural or functional evidence of disease with any Tg and TgAb levels

Based on the last follow-up result, the recruited patients were divided into two groups: the ER group, including patients with ER, and the NER group, including patients with IDR, BIR, and SIR.

### Statistical analysis

Statistical analysis was performed by using SPSS 26.0 (Chicago, Illinois, USA). Categorical variables were expressed as counts with percentages, and group differences were assessed by the chi-squared test. Continuous variables were presented as medians with interquartile ranges or mean with standard deviation. The Mann–Whitney U-test was used to examine the non-normally distributed continuous variables. Spearman’s correlation was used for the correlation analysis between sTg and pa-TgAb prior to the initial RAIT. Receiver operating characteristic (ROC) curve analysis was utilized to assess the predictive value. Logistic regression was performed to obtain the independent prognostic factors for ER. Analysis of DFS was performed by using the Kaplan–Meier method, and the groups were compared by log-rank tests. The difference between the two groups was defined as statistically significant when *p*-values <0.05.

## Results

### Baseline clinical characteristics for enrolled patients

**Figure 1:**
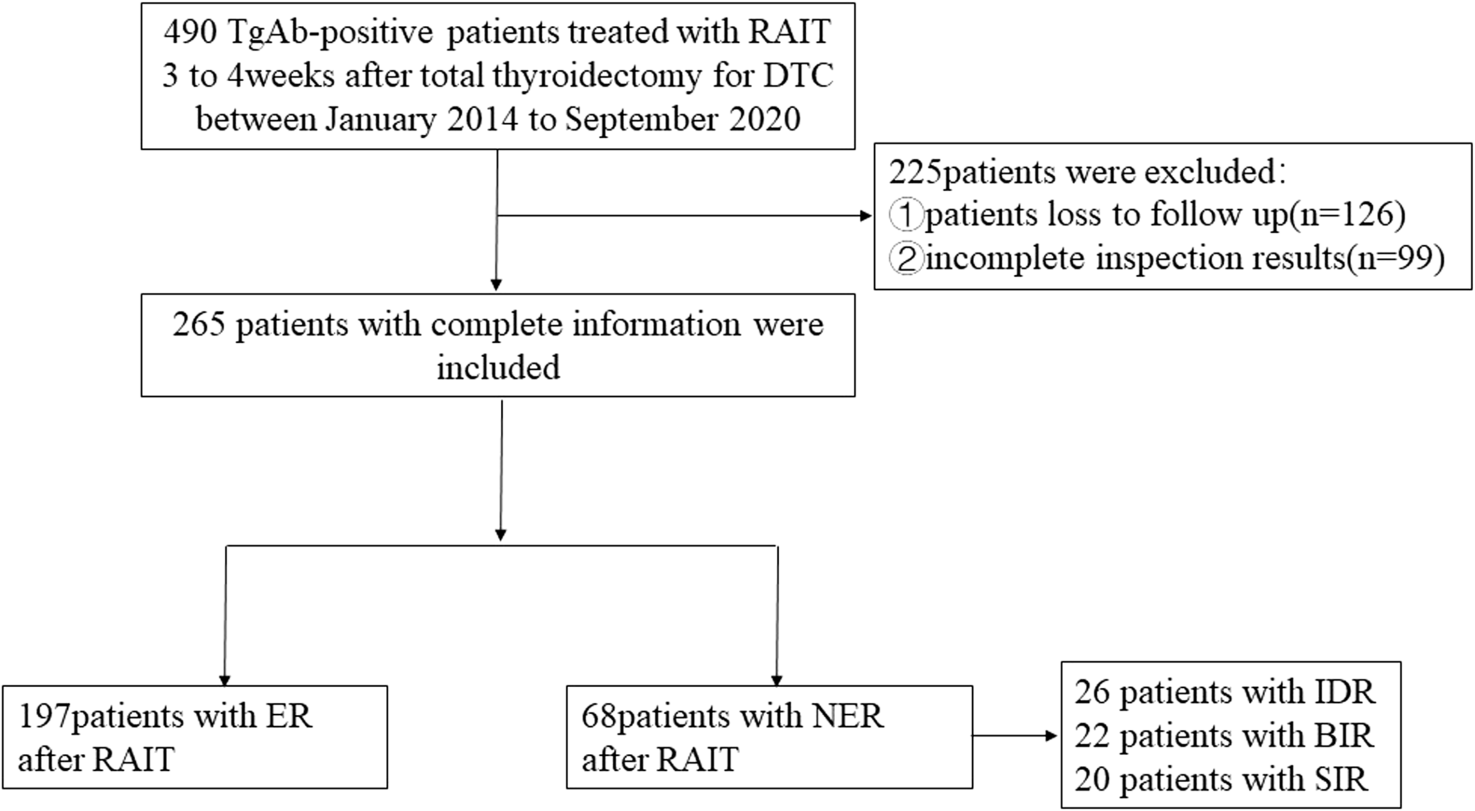
Flowchart of patient inclusions and exclusions in the study.

**Figure 1** shows the flowchart of this study. A total of 265 TgAb-positive DTC patients were enrolled based on the inclusion and exclusion criteria. The median follow-up time was 48 months. There were 262 cases of papillary thyroid cancer (PTC) and 3 cases of follicular thyroid cancer (FTC). The PTC cases included 242 classic PTC cases, 3 tall cell variants, 3 follicular variants, 8 hobnail variants, 6 oncocytic variants, and 3 diffuse sclerosing variants. All of the cases received TT, and 112 cases (42.26%) received modified radical neck dissection (MRND). There were 187 cases with a metastatic lymph node (LN) number greater than 5 and 29 cases with an extra-nodal extension in this part of cases. For cases with a metastatic LN number less than 5 (78/265), there were totally 6 cases with an extra-nodal extension, 42 cases with a high T stage (T3–T4), and 30 cases with the maximal diameter of the metastatic LN greater than 3 cm.

### Comparison between the clinicopathological features of the two groups

**Figure 2:**
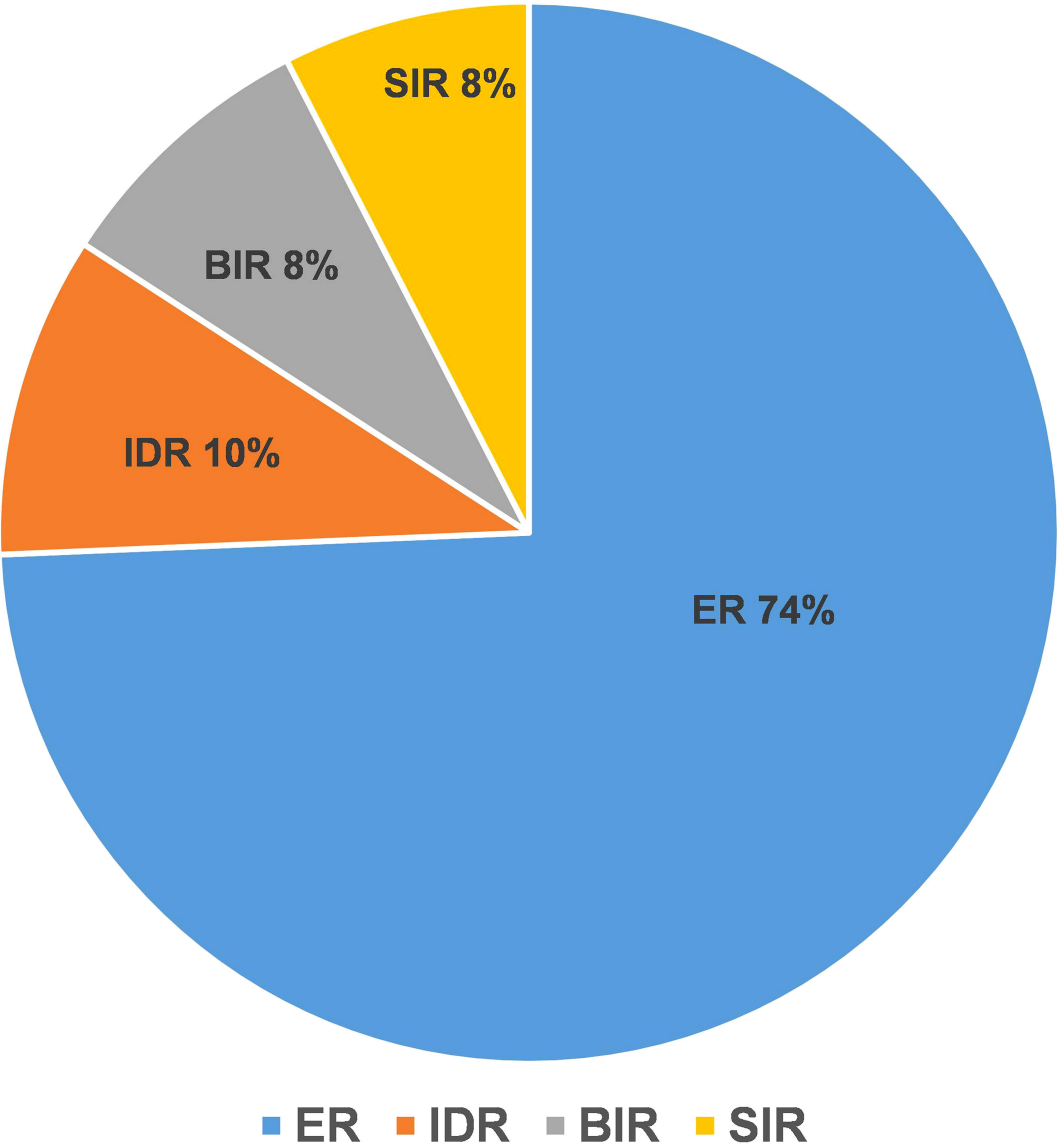
The therapeutic response to RAIT of 265 patients.

The ER group included 197 patients, and the NER group included 68 patients. In the NER group, there were 26 patients with IDR, 22 patients with BIR, and 20 patients with SIR (**Figure 2**). There were a total of 4 cases with distant metastasis, and for all of them, the distant metastasis organ was the lung. Correlation analysis showed a negative correlation between sTg and pa-TgAb (r = −0.157, *p* = 0.011). Maximal tumor diameter, tumor with ETE, bilateral or unilateral primary lesion, multifocality, preop-TgAb, pa-TgAb, sTg × pa-TgAb, initial RAIT dose, N stage, and surgical extent (modified radical neck dissection or not) showed significant differences between the ER group and NER group (all p-values <0.05), while sex, age at diagnosis, primary lesion, HT, tumor stage, and risk stratification of recurrence showed no statistically significant differences (*p*-values >0.05 for all). A comparison of clinicopathological characteristics on the efficacy of RAIT in the 265 patients is summarized in **Table 2**.

**Table 2:** Comparison of clinicopathological characteristics on the efficacy of RAIT.

Characteristics	ER group (n = 197)	NER group (n = 68)	χ^2^/Z-test	*p*-value
Gender			0.916	0.339
Men	23(11.68%)	11(16.18%)		
Women	174(88.32%)	57(83.82%)		
Age at diagnosis			1.573	0.210
<55 years	174	56		
≥55 years	23	12		
Maximal tumor diameter	1.70(1.00,2.20)	1.50(1.00,2.16)	−2.568	0.010*
Primary lesion				
Unilateral	119(60.41%)	31(45.59%)	4.519	0.034*
Bilateral	78(39.59%)	37(54.41%)		
Multifocality			3.898	0.048*
Yes	61	30		
No	133	37		
Unclear	3	1		
HT				
Yes	122(61.93%)	37(54.41%)	1.190	0.275
No	75(38.07%)	31(45.59%)		
ETE				
Yes	124(62.94%)	54(79.41%)	6.216	0.013*
No	73(37.06%)	14(20.59%)		
MRND			4.273	0.039*
Yes	76	36		
No	121	32		
Preop-TgAb (IU/ml)	402.10(251.40,762.85)	588.65(358.20,2,127.00)	−2.852	0.004*
pa-TgAb (IU/ml)	303.50(157.70,489.45)	454.00(258.38,823.13)	−3.931	0.000*
sTg × pa-TgAb	55.66(16.03,339.92)	265.82(46.61,1,441.60)	−4.270	0.000*
T stage			7.092	0.131
Tx	3(1.52%)	1(1.47%)		
T1	109(55.33%)	30(44.11%)		
T2	17(8.63%)	13(19.12%)		
T3	39(19.80%)	11(16.18%)		
T4	29(14.72%)	13(19.12%)		
N stage			19.105	0.000*
N0	31(15.74%)	1(1.47%)		
N1a	60(30.46%)	11(16.18%)		
N1b	106(53.80%)	56(82.35%)		
Risk stratification of recurrence			0.013	0.908
Intermediate risk	129(65.48%)	44(64.71%)		
High risk	68(34.52%)	24(35.29%)		
Initial RAIT dose (mCi)	100(100,120)	120(100,150)	−5.136	0.000*

HT, Hashimoto’s thyroiditis; ETE, extrathyroidal extension; MRND, modified radical neck dissection; preop-TgAb, preoperative TgAb; pa-TgAb, pre-ablation TgAb; RAI, radioiodine therapy; *p-value <0.05.

### ROC curves for predicting NER

**Figure 3:**
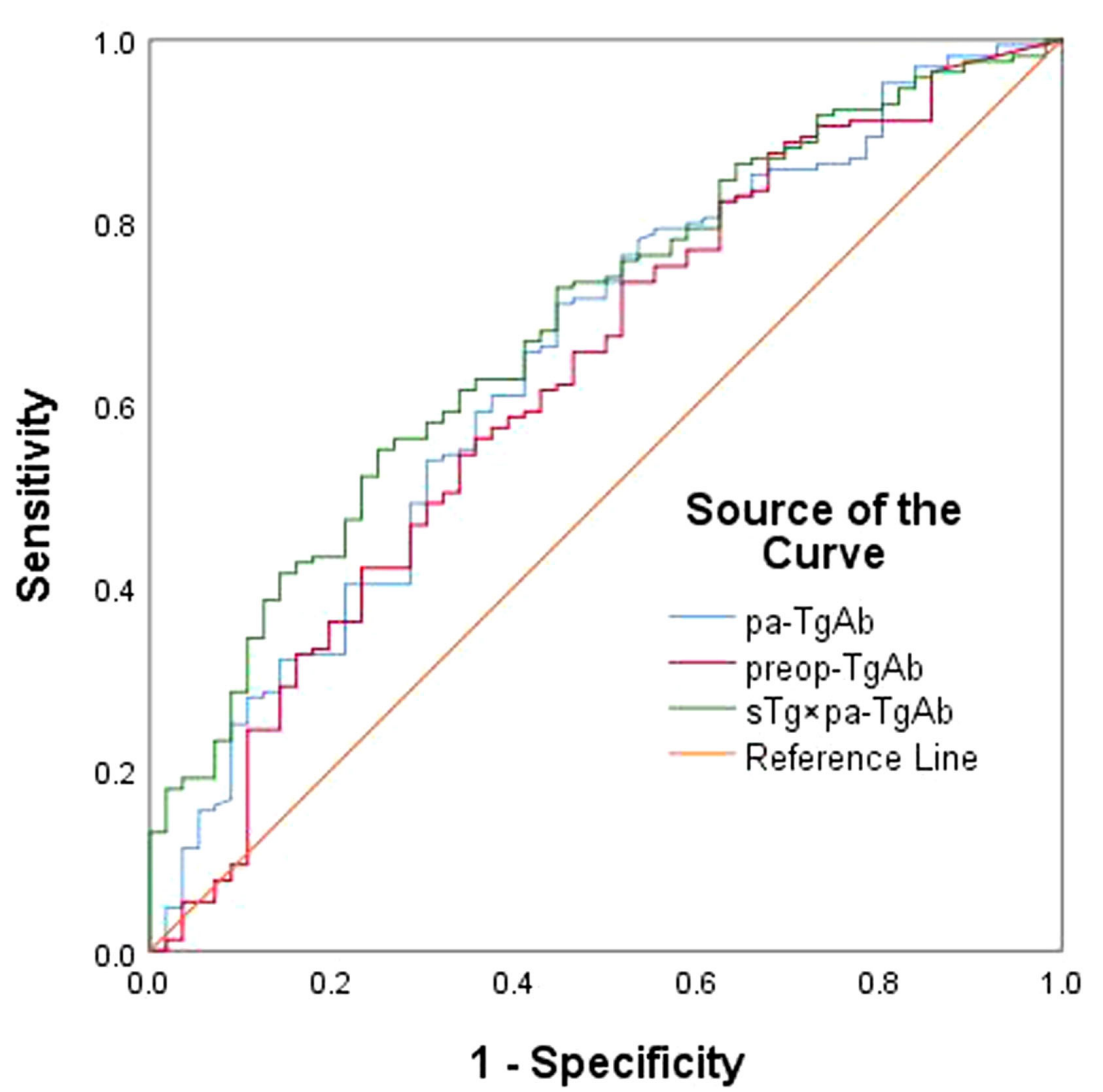
ROC curves for preop-TgAb, pa-TgAb, and sTg × pa-TgAb before initial RAIT for predicting ER.

We performed an ROC curve analysis on preop-TgAb, pa-TgAb, and sTg × pa-TgAb. **Figure 3** shows that the areas under the curve (AUCs) of preop-TgAb, pa-TgAb, and sTg × pa-TgAb were 0.627, 0.650, and 0.687, respectively. **Table 3** shows that the corresponding cutoff values were 724.25 IU/ml, 424.00 IU/ml, and 59.73, respectively.

**Table 3:** Cutoff values for predicting ER with sensitivity and specificity.

Characteristics	Cutoff value	Sensitivity (%)	Specificity (%)	PPV (%)	NPV (%)	AUC (95% CI)	*p*-value
Preop-TgAb (IU/ml)	724.25	73.4	48.2	38.03	81.29	0.628(0.541–0.715)	0.004
pa-TgAb (IU/ml)	424.00	71.6	54.4	39.78	81.98	0.650(0.566–0.734	0.001
sTg × pa-TgAb	59.73	51.8	73.5	34.48	85.00	0.690(0.613–0.767)	0.000

AUC, area under the curve; PPV, positive predictive value; NPV, negative predictive value; CI, confidence interval.

### Univariate and multivariate logistic regression analysis

The univariate logistic regression analysis showed that maximal tumor diameter, bilateral or unilateral primary lesion, tumor with ETE, multifocality, surgical extent (modified radical neck dissection or not), preop-TgAb (≥724.25 IU/ml), pa-TgAb (≥424.00 IU/ml), sTg × pa-TgAb (≥59.73), N stage, and initial RAIT dose were significantly different between the ER group and NER group. On the other hand, sex, age at diagnosis, HT status, and risk stratification of recurrence were not significantly related to ER. Then, we further analyzed the significantly different factors with multivariate logistic regression analysis. We found that pa-TgAb (*p* = 0.034), sTg × pa-TgAb (*p* = 0.025), N stage (*p* = 0.006), and initial RAIT dose (*p* = 0.000) were independent risk factors for predicting NER. **Table 4** shows the details of the univariate and multivariate logistic regression analysis results.

**Table 4:** Univariate and multivariate logistic analysis of therapeutic effect.

Characteristics	Univariate logistic analysis		Multivariate logistic analysis	
	OR (95% CI)	*p-*value	OR (95% CI)	p-value
Gender	0.685(0.315–1.492)	0.341		
Age at diagnosis (<55 years vs. ≥55 years)	1.621(0.758–3.467)	0.213		
Maximal tumor diameter	1.426(1.029–1.976)	0.033	1.005(0.632–1.598)	0.982
Primary lesion	1.821(1.044–3.176)	0.035	1.563(0.697–3.506)	0.279
Multifocality	0.566(0.320–0.999)	0.050	0.775(0.346–1.737)	0.536
HT	0.734(0.420–1.281)	0.276		
ETE	0.481(0.253–0.914)	0.025	0.642(0.276–1.494)	0.304
MRND (yes vs. no)	0.558(0.320–0.973)	0.040	0.892(0.385–2.064)	0.789
Preop-TgAb (IU/ml) (<724.25 vs. ≥724.25)	2.388(1.277–4.467)	0.006	0.798(0.286–2.223)	0.665
pa-TgAb (IU/ml) (<424.00 vs. ≥424.00)	3.189(1.803–5.640)	0.000	2.856(1.080–7.551)	0.034*
sTg × pa-TgAb (<59.73 vs. ≥59.73)	2.922(1.593–5.362)	0.001	2.455(1.118–5.391)	0.025*
N stage	3.281(1.843–5.842)	0.000	3.526(1.433–8.676)	0.006*
Risk stratification of recurrence	1.035(0.581–1.844)	0.908		
Initial RAIT dose (mCi) (≤100 vs. >100)	4.689(2.603–8.445)	0.000	3.931(1.894–8.158)	0.000*

OR, odds ratio; CI, confidence interval. * means p-value <0.05.

### Kaplan–Meier analysis of DFS

**Figure 4:**
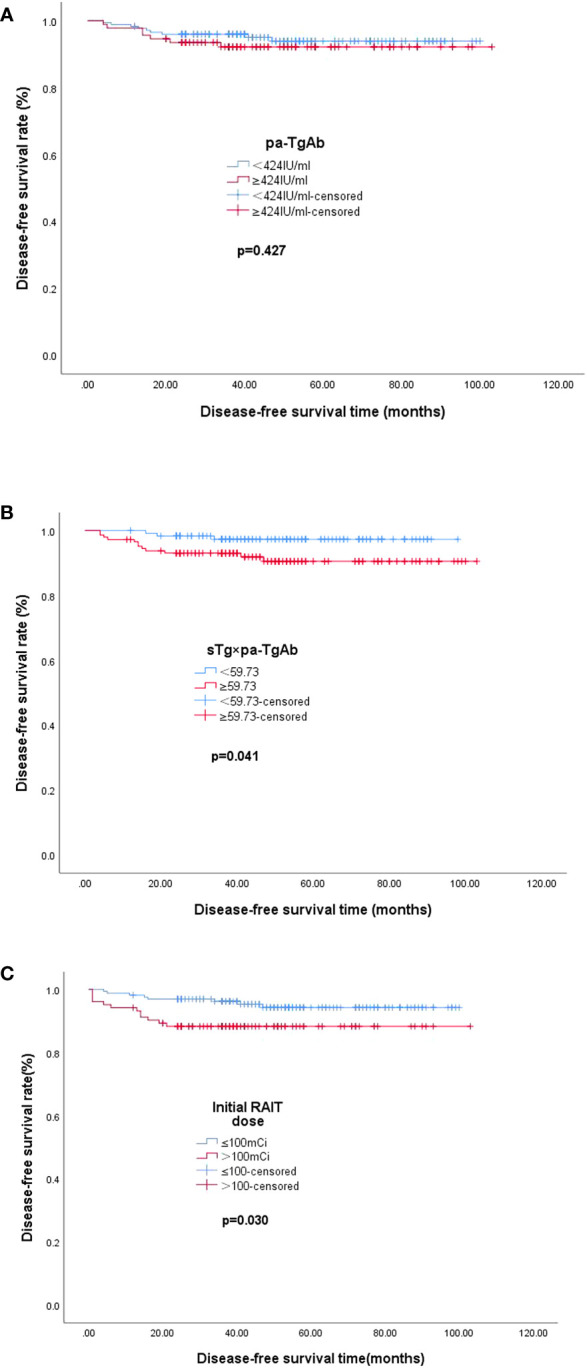
Median DFS. **(A)** Median DFS in the group of patients with pa-TgAb < 424 IU/ml (n = 173) or ≥424 IU/ml (n = 92). The median DFS was 49.68 months with pa-TgAb < 424 IU/ml and 48.72 months with pa-TgAb ≥ 424 IU/ml (*p* = 0.427). **(B)** Median DFS in the group of patients with sTg × pa-TgAb < 59.73 (n = 120) or ≥59.73 (n = 145). The median DFS was 50.27 months with sTg × pa-TgAb < 59.73 and 48.59 months with sTg × pa-TgAb ≥ 59.73 (*p* = 0.041). **(C)** Median DFS in the group of patients with initial RAIT dose ≤100 mCi (n = 162) or >100 mCi (n = 103). The median DFS was 50.50 months with initial RAIT dose ≤100 mCi and 38.00 months with initial RAIT dose >100 mCi (*p* = 0.030).

The median DFS values of the patients with pa-TgAb ≥ 424.00 IU/ml and the patients with pa-TgAb < 424.00 IU/ml were not significantly different (48.72 months vs. 49.68 months, *p* = 0.427). The median DFS in the sTg × pa-TgAb < 59.73 patients and initial RAIT dose ≤100 mCi was significantly longer than that of the patients with sTg × pa-TgAb ≥ 59.73 (50.27 months vs. 48.59 months, *p* = 0.041) and initial RAIT dose >100 mCi (50.50 months vs. 38.00 months, *p* = 0.030) (**Figure 4**).

## Discussion

In recent years, the incidence of DTC has increased globally (23, 24). As a specific tumor marker for long-term follow-up in DTC, Tg can predict DTC persistence, distant metastasis, or recurrence. However, the Tg level can be affected by TgAb, which makes Tg measurement unreliable and limits the role of Tg in prognosis in DTC patient follow-up. Therefore, TgAb has gained increasing attention as an essential indicator for DTC surveillance. Prior studies of the clinical importance of TgAb in DTC have typically focused on the sequential changes in TgAb titers (12–16). Many studies have reported that the stable or rising trend of TgAb titers after TT is an effective risk factor for predicting persistent or recurrent disease (25–27). Pan et al. indicated that TgAb affected sTg measurements even if they were negative. They reported a novel negative correlation between TgAb and Tg levels, suggesting that the sTg × TgAb product could be used to predict prognosis in patients with DTC (17). However, the majority of the patients in that study were TgAb-negative patients (321/409). Therefore, the present study explored the prognostic value of Tg × pa-TgAb in TgAb-positive DTC patients since these patients are not rare and account for almost 25% of DTC patients (6).

We enrolled 265 TgAb-positive DTC patients in the present study. The results revealed that preop-TgAb, pa-TgAb, and sTg × pa-TgAb were significantly correlated with an excellent response to RAIT. Some previous reports indicated that high preoperative TgAb was an independent predictor of nodal metastases in DTC patients (28, 29). Some studies (18, 30) indicated that pa-TgAb showed significant differences between different efficacy groups. Pan et al. (17) indicated that sTg × pa-TgAb could be used to predict prognosis in patients with DTC. These results were similar to those of our present study. Our data also suggested that maximal tumor diameter, primary lesions, tumor with ETE, and N stage were significantly different between the ER group and NER group, which is consistent with previous reports (14, 31).

To confirm the predictive value of the three factors, we generated ROC curves for further analysis. The results demonstrated that the cutoff values of preop-TgAb, pa-TgAb, and sTg × pa-TgAb were 724.25 IU/ml, 424.00 IU/ml, and 59.73, respectively. According to the cutoff values, we divided the preop-TgAb, pa-TgAb, and sTg × pa-TgAb products into two groups. The multivariate logistic regression analyses indicated that pa-TgAb, sTg × pa-TgAb, and N stage were independent risk factors for NER. The risk of NER increases by 2.856-fold for each 1 kU/L increase in the level of pa-TgAb and by 2.455-fold for sTg × pa-TgAb. As mentioned before, Pan et al. (17) reported that the sTg × TgAb product cutoff value in the TgAb-positive group was 466.36, which was much higher than that in our study. One reason for the difference may be that the definition of TgAb positivity was different, which in our study was 40 IU/ml before the initial RAIT, while in Pan et al.’s study, it was 115 IU/ml. This difference led to the patients’ demographic natures being different. The other reason may be the differences in sample size in the studies, in which the sample size of TgAb-positive patients in Pan et al.’s study was smaller than that in our study (88 vs. 265).

It has been reported that the RAIT dose was a vital factor affecting the therapeutic effect of RAIT ablation. Previous research studies indicated that a higher RAIT dose was an independent risk factor for unsuccessful ablation (17). The results of the present study were similar to those of previous studies. We found that unsuccessful ablation increased by 3.931-fold for every increase of 1 mCi in the RAIT dose.

In addition, we found that the median DFS of patients with sTg × pa-TgAb < 59.73 was longer than that of patients with sTg × pa-TgAb ≥ 59.73 (50.27 months vs. 48.59 months, *p* = 0.041), which confirmed the prognostic value for TgAb-positive patients. However, the results of the Kaplan–Meier analysis showed similar median DFS values for patients with pa-TgAb < 424 IU/ml and TgAb ≥ 424 IU/ml.

Nonetheless, there were still some limitations in our study. On the one hand, this study was a retrospective single-center study, and the sample size was relatively small; thus, selection bias cannot be avoided. Therefore, further prospective multicenter large-scale studies are required for validation. On the other hand, longer periods of follow-up are required to confirm the current findings.

In summary, we found that the sTg and pa-TgAb product can predict the efficacy as well as prognosis of RAIT for TgAb-positive DTC patients. Since sTg interferes with TgAb before RAIT in these patients, the product of sTg and pa-TgAb can be an effective clinical reference indicator to recognize NER patients early, which has a very positive and important role in optimizing treatment, improving prognosis, and reducing the burden of patients.

## Data availability statement

The original contributions presented in the study are included in the article/supplementary material. Further inquiries can be directed to the corresponding author.

## Ethics statement

The studies involving humans were approved by the Ethics Committee of the Affiliated Hospital of Qingdao University. The studies were conducted in accordance with the local legislation and institutional requirements. The participants provided their written informed consent to participate in this study.

## Author contributions

NH wrote the main manuscript text. CL, JL, and CW helped draft the manuscript. XW helped revise the manuscript. ZZ, YZ, XL, and ZS coordinated the research and analyzed the data. GW, ZW, and FL performed the methodology and formal analysis. All authors contributed to the article and approved the submitted version.
